# Supplementary material for: Complexome profiling on the Chlamydomonas lpa2 mutant reveals insights into PSII biogenesis and new PSII associated proteins
Source: J Exp Bot. 2021 Aug 26;73(1):245–62. doi: 10.1093/jxb/erab390 (PMC8730698; doi:10.1093/jxb/erab390)
Supplement: erab390_suppl_Supplementary_Dataset_S1 [file erab390_suppl_supplementary_dataset_s1.zip › Supplemental Dataset 1 - Excel List and all profiles/plots/_Cre08.g381702.html]

Trivial name:   
  
Euclidean distance: 13549.93  
Mean Intensity (WT): 1119.13  
Mean Intensity (Mut): 558.50  
Distance: 12.11  
  
MapMan: TCA / organic transformation.other organic acid transformations.misc;secondary metabolism.N misc.betaine;amino acid metabolism.synthesis.central amino acid metabolism.GABA.SSADH  
  
p value of intensity sums Welch test: 0.3486
